# Application of Hyperspectral Imaging as a Nondestructive Technology for Identifying Tomato Maturity and Quantitatively Predicting Lycopene Content

**DOI:** 10.3390/foods12152957

**Published:** 2023-08-04

**Authors:** Chunxia Dai, Jun Sun, Xingyi Huang, Xiaorui Zhang, Xiaoyu Tian, Wei Wang, Jingtao Sun, Yu Luan

**Affiliations:** 1 School of Electrical and Information Engineering, Jiangsu University, Xuefu Road 301, Zhenjiang 212013, China; 2 School of Food and Biological Engineering, Jiangsu University, Xuefu Road 301, Zhenjiang 212013, Chinatianxiaoyucau@163.com (X.T.); 3 College of Engineering, China Agricultural University, Beijing 100083, China; 4 School of Food Science and Technology, Shihezi University, Shihezi 832000, China; 5 Zhenjiang Food and Drug Supervision and Inspection Center, Zhenjiang 212004, China

**Keywords:** hyperspectral imaging technology, tomato maturity, lycopene content, classification, regression model, visualization

## Abstract

Maturity is a crucial indicator in assessing the quality of tomatoes, and it is closely related to lycopene content. Using hyperspectral imaging, this study aimed to monitor tomato maturity and predict its lycopene content at different maturity stages. Standard normal variable (SNV) transformation was applied to preprocess the hyperspectral data. Then, using competitive adaptive reweighted sampling (CARS), the characteristic wavelengths were selected to simplify the calibration models. Based on the full and characteristic wavelengths, a support vector classifier (SVC) model was developed to determine tomato maturity qualitatively. The results demonstrated that the classification accuracy using the characteristic wavelength led to the obtention of better results with an accuracy of 95.83%. In addition, the support vector regression (SVR) and partial least squares regression (PLSR) models were utilized to predict lycopene content. With a coefficient of determination for prediction (R^2^_P_) of 0.9652 and a root mean square error for prediction (RMSEP) of 0.0166 mg/kg, the SVR model exhibited the best quantitative prediction capacity based on the characteristic wavelengths. Following this, a visual distribution map was created to evaluate the lycopene content in tomato fruit intuitively. The results demonstrated the viability of hyperspectral imaging for detecting tomato maturity and quantitatively predicting the lycopene content during storage.

## 1. Introduction

Tomatoes have become one of the world’s most important fruits [1]. China is currently the world’s largest producer and exporter of tomatoes. Tomatoes significantly impact the development of China’s agricultural trade economy. They are extremely popular with consumers due to their sweet and sour taste and rich nutritional value [2]. In addition, they are abundant in vitamins and minerals [3,4]. Lycopene in tomatoes has been receiving a lot of attention lately, because it can capture free radicals in the body, thereby protecting cells, and has various functions, such as preventing various cancers and reducing atherosclerosis incidence [5,6]. Tomatoes are generally harvested at the early maturity stage to increase storage life [7,8]. Green ripe tomatoes picked in the same batch do not ripen at a synchronized pace during storage, and some fruits may ripen first and become unsuitable for long-term storage. Nonetheless, due to tomatoes’ characteristics, those with a high degree of maturity not only have poor storage performance but also induce the surrounding fruit to enter the stage of respiratory hopping, thereby diminishing their overall quality [9,10]. Therefore, in the tomato storage process, it is of great practical importance to rapidly identify tomato ripeness, which not only can guide people to consume tomatoes of high maturity and good quality, but also can promptly remove tomatoes unsuitable for storage.

Artificial sensory evaluation and physical and chemical analysis are the most common methods used for determining the quality of tomatoes. Among them, sensory evaluation has strict requirements for assessing personnel and the environment, and assessors can easily influence the evaluation results [11]. On the other hand, physical and chemical tests rely on expensive and sophisticated instruments and lengthy procedures. Moreover, they may harm the samples. Therefore, it is necessary to develop a method that is objective, intelligent, and nondestructive for determining the quality of tomatoes, which will be useful for producers, processors and distributors to determine the quality of tomatoes. Physical and chemical indicators of tomato quality include hardness, and lycopene, vitamin C, soluble solid, and titratable acid content, among others. Among these, lycopene content is a crucial indicator frequently employed to characterize the quality of tomatoes. 

Electronic nose [12,13], near-infrared spectroscopy (NIRS) [14,15], and machine vision [16,17] are notable intelligent detection technologies that are used to assess tomato quality. Based on the volatile odor substances and colors of samples at different maturation stages, they detect maturity. However, these techniques do not provide the most accurate physical and chemical index predictions. NIRS is a nondestructive tool for analyzing the quality of multiple foods’ raw material components [18]. However, NIRS spectral assessments of relatively small point source measurements do not include spatial information, which is crucial for many food inspection applications [19].

Hyperspectral imaging technology is a technical fusion technology of spectral analysis technology and image processing technology. It was created in the 1980s. It combines traditional two-dimensional imaging and spectroscopy technology with ultra-multi-band high spectral resolution and map uniformity [20]. Compared with single NIRS or machine vision technology, it provides more comprehensive information. It has been researched in fruit quality testing and other tests. It can simultaneously collect fruits’ appearance and spectral information to evaluate their internal and external quality. Rajkumar et al. [21] used hyperspectral imaging for the prediction of the total soluble solids, moisture content and firmness of banana fruits at three different temperatures. The results showed that the best effect was obtained using the multiple linear regression method with coefficients of determination of 0.85, 0.87, and 0.91, respectively. Sun et al. [22] used a near-infrared hyperspectral imaging system to predict the sweetness and hardness of melons. Among the three prediction models, the PLSR model was the best for prediction using either the full spectrum or the most important spectrum. Li et al. [23] studied cherries at different stages using NIR hyperspectral imaging technology. GA-MLR models obtained the best performance in predicting both the soluble solids content and PH. The R^2^p for soluble solids content was 0.863, and RMSEP was 1.210%. The R^2^p for PH was 0.819, and RMSEP was 0.057%. The linear discriminant analysis method was used to classify the cherry maturity stage, and the correct classification rate was 96.4%. 

Due to variations in tomato maturity, their physical and chemical properties change, as do their light reflection and projection capabilities. Therefore, based on prior studies, hyperspectral image technology was used to distinguish tomato maturity and quantitatively predict the corresponding indicators qualitatively.

Hyperspectral imaging’s ability to provide spatial information of attributes makes it most ideal for this study. In this paper, the nondestructive detection technology of hyperspectral imaging is used to qualitatively identify tomato maturity during storage and quantitatively predict the lycopene content of tomatoes. It can serve as a guide for determining the optimal tomato consumption period.

## 2. Materials and Methods

### 2.1. Tomato Samples

Tomatoes of the “Jinpeng” variety were purchased from a greenhouse in Zhenjiang, China. All tomatoes were harvested by hand when they were still at the green maturity stage. Overall, 144 tomatoes weighing 180 ± 20 g were selected. After being numbered, the tomatoes were stored in a constant-temperature humidity room at approximately 20 °C and 85% relative humidity. Following the United States Department of Agriculture’s grading standard for fresh tomatoes, samples are classified into different stages based on their appearance description in Table 1 [24].

### 2.2. Hyperspectral Image System and Data Acquisition

The hyperspectral imaging system used in this study consisted primarily of a spectrometer (ImSpector V10E, Spectral Imaging Co., Ltd., Oulu, Finland), a CCD camera with a detector size of 1024 × 478 pixels, a lens (OLE23, Specim Ltd., Oulu, Finland), a 390 nm UV light source (UBerLEDTM 50, Illumination Technology, Inc., New York, NY, USA), and a stepper motor and a computer [25]. Before acquiring hyperspectral images, the instrument had to be preheated for 30 min. Then, the end of the tomato sample’s pedicle was placed horizontally on the platform, and the lens distance was set to 400 mm. The camera exposure time was 10.5 ms, and the translation platform’s speed was 5.2134 mm/s. One hundred forty-four hyperspectral images were obtained in total. The collected spectral range was 480.80–1002.20 nm, with a total of 411 bands.

### 2.3. Spectra Extraction

Raw hyperspectral images, *Iraw*, were calibrated using the following expression to eliminate the effects of irregular light source intensities and the dark current.

*Ical* = (*Iraw* − *Idark*)/(*Iwhite* − *Idark*) (1)

where *Ical* indicates the calibrated image, *Iraw* means the raw image, *Idark* represents the dark reference image, and *Iwhite* denotes the white reference image.

**Figure 1:**
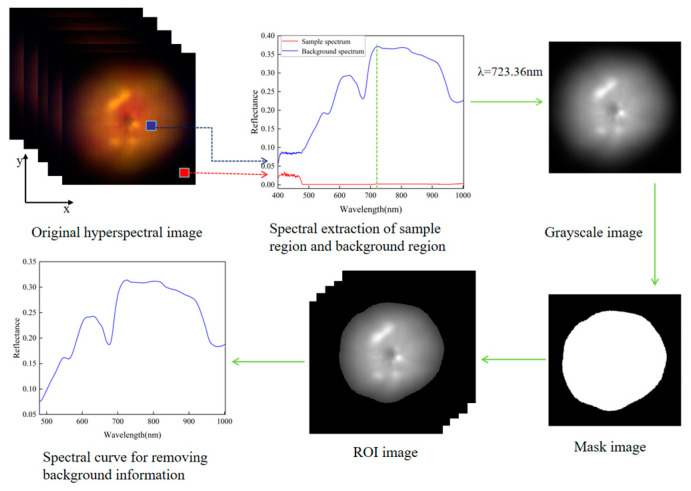
Specific steps of spectral extraction.

After calibrating the hyperspectral image, threshold segmentation was applied to determine the region of interest (ROI) and remove background information. Figure 1 demonstrates the extraction procedures for spectral data. First, the mask image was generated using a gray image at a high-fluorescence-intensity band (723.36 nm) and a threshold of 0.25. The ROI was then constructed using the mask to eliminate background information from the hyperspectral image. Next, the mean spectral value of all pixels within the ROI was calculated to represent the spectral data for each tomato sample [26]. Since the hyperspectral data of samples before 480 nm were greatly affected by hardware noise, it was impossible to eliminate the wavelengths with noise. The final spectral data were obtained for 411 wavelengths, with a wavelength range of 480.80–1002.20 nm.

### 2.4. Determination of the Lycopene Content

The lycopene content was determined using the agricultural standard NY/T 1651–2008 “Determination of lycopene in vegetables and products-HPLC”. The HPLC conditions were as follows. The detection wavelengths were 472 nm. The column was made of C18 stainless steel and measured 250 nm in length, 4.6 mm in diameter, and 5 mm in particle size. The mobile phase was a 4:15:1 volume mixture of methanol, acetonitrile, and dichloromethane; all three reagents were chromatographically pure. The flow rate was 1.0 mL and the injection volume was 10 µL.

We first constructed a standard curve from lycopene standards to determine the lycopene content of tomato samples. The preparation of the lycopene standard solution consisted primarily of the following steps. Briefly, 10 mg of lycopene standard product with a purity of ≥98% was weighed, and chromatographically pure dichloromethane was used as the solvent to prepare a lycopene standard product with a concentration of 100 mg/L. Subsequently, gradient dilution with dichloromethane was used to prepare lycopene standard solutions with 1, 5, 10, 20, and 30 mg/L concentrations, A 0.45 μm microporous membrane filtered the standard solution before it was transferred to a brown liquid phase vial. The area of the peak was then determined using a high-performance liquid chromatograph.

### 2.5. Data Analysis

#### 2.5.1. Data Preprocessing

Spectral pre-processing is an important step in establishing a spectral detection algorithm in order to reduce errors due to external factors and random noise, and baseline changes [27]. Additionally, influencing the spectrum are factors such as the scattering of the object’s surface and the alteration of the optical path. For this reason, SNV transformation was chosen and implemented to preprocess the black-and-white calibrated hyperspectral data.

SNV is mainly used to correct each spectrum for errors due to particle scattering, using the principle that the absorbance values at each wavelength of the spectrum satisfy a certain distribution. The SNV transform is corrected by subtracting the mean value of the spectrum from the original spectrum and dividing it by the standard deviation of the original spectrum. The formula is shown as follows:

(2) $$X_{SNV}=\frac{X_{K}-\bar{X}}{\sqrt{\sum_{1}^{N}{\left(X_{K}-\bar{X}\right)}^{2}/(N-1)}}$$

where $X_{SNV}$ means the spectrum corrected by SNV, $X_{K}$ indicates the original spectrum, $\bar{X}$ represents the mean value of the spectrum, and N denotes the number of wavelengths.

#### 2.5.2. Feature Selection

Since hyperspectral data consist of hundreds of continuous wavelengths, the data volume is large, and the adjacent wavelengths are highly correlated and redundant [28]. Therefore, feature selection is essential for simplifying the model and improving detection efficiency. CARS is a feature selection method based on Monte Carlo sampling (MCS) and PLSR regression coefficients that selects the optimal subset of wavelengths by mimicking the “survival of the fittest” principle [29]. In this study, CARS was used to select features from full spectra to develop a simple and efficient prediction model.

In the CARS, each variable is a single individual, and in each iteration, the highly adaptive individuals are retained and the weakly adaptive individuals are eliminated. The process of eliminating individuals relies on the exponential decreasing function (EDF) and adaptive reweighted sampling (ARS), which retains variables with large absolute values of the regression coefficients and excludes those with small absolute values of the regression coefficients in each modeling process. Using the interactive validation method, the variables in the subset with the minimum RMSECV are selected as the feature variables. The main steps of the CARS are as follows. 

Firstly, the calibration set and prediction set are determined from the sample set in a certain proportion based on the MCS method. The PLSR model is built, and the regression coefficients corresponding to each variable are calculated. 

Secondly, the EDF is used to delete the variables with small absolute values of regression coefficients; on the basis of this operation, the variables are further eliminated with the help of ARS, and the above steps are repeated to form an iterative process. 

Finally, the RMSECV of the selected subset of variables is calculated, and the variables contained in the subset with the minimum RMSECV are taken as the selected feature variables. 

### 2.6. Model Establishment and Evaluation

SVM is a method based on statistical principles and follows the principle of structural risk minimization. It achieves the purpose of pattern recognition through the supervised learning of samples. SVM not only has a simple computation and a short training time, but also has good generalization performance and robustness [30]. SVM can be used for both data classification and regression analysis. Using a nonlinear model, a support vector classifier (SVC) can handle high-dimensional data. In addition, its accuracy is superior to that of other machine learning algorithms [31]. 

The basic idea of the SVR algorithm is to create an optimal hyperplane by using the training sample, and minimize the total deviation of the sample points from the hyperplane by approximating the samples to the hyperplane. The kernel function is used to achieve nonlinear mapping from the input space to the high-dimensional space, so as to realize linear regression and improve the learning effect [32]. SVM uses different kernel functions and obtains different support vector machine algorithms. This paper optimizes the parameters c and g using grid optimization algorithm search methods and five-fold cross-validation methods.

PLSR is a traditional multivariate linear statistical analysis method that maps multidimensional data onto orthogonal latent variable (LV) factors. The minimum predicted residual error sum of squares was used to determine the optimal number of LVs [33].

SVC was used to classify the tomatoes. Utilizing PLSR and SVR, a correlation between hyperspectral data and lycopene content was established. The accuracy and precision of the model were measured using statistical measures such as the coefficient of determination for calibration (R^2^_C_), R^2^_P_, root mean squared errors of cross-validation (RMSECV) and RMSEP. MATLAB 2020a was used to perform the data analysis, with Windows 11 as the operating system.

## 3. Results and Discussion

### 3.1. Lycopene Analysis

**Figure 2:**
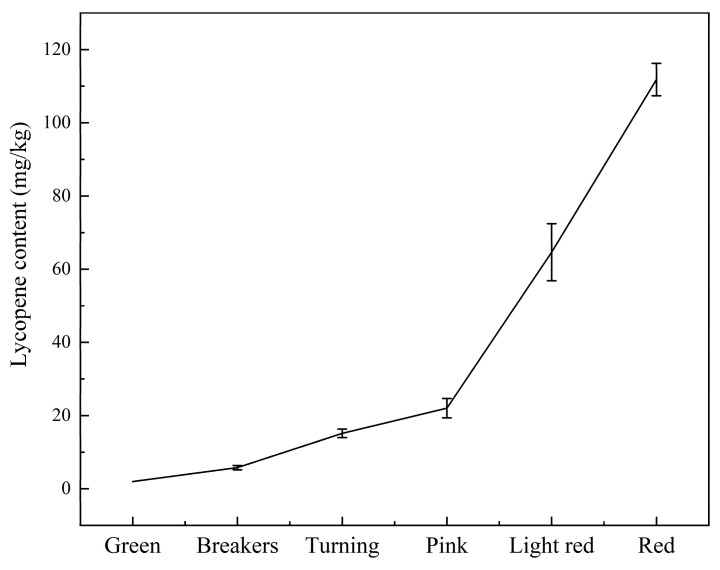
Lycopene contents of tomatoes at different maturity stages.

Lycopene content is a relatively intuitive indicator of tomato maturity. Due to variations in lycopene content, the color of a tomato also varies. Figure 2 depicts the mean and standard deviation of lycopene content during storage. It is evident from the figure that the lycopene content of tomato samples increased from 1.97 mg/kg to 111.82 mg/kg. Almost no lycopene was present during the green stage, and lycopene synthesis did not begin until the breaker stage. In subsequent stages, the lycopene content also increased.

### 3.2. Spectral Feature Analysis

**Figure 3:**
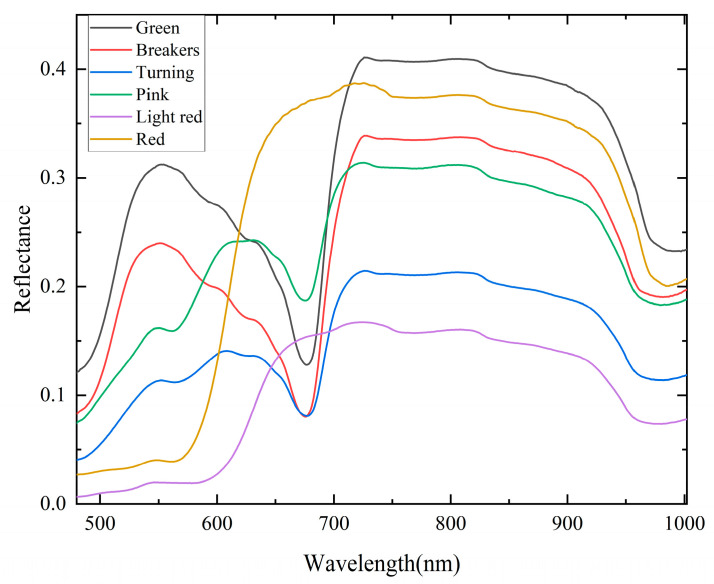
Average spectral curves of tomatoes at different maturity stages.

Figure 3 depicts the average spectral curves in the range 480.80–1002.20 nm for tomatoes at various maturity stages. In the range 480.80–700.35 nm, the trends of the spectral reflectance were different, whereas they were similar in the range 723.36–969.92 nm. The significant difference in the visible range was likely caused by the color changes of tomatoes at various maturity stages [34]. The wave trough near 670 nm was associated with chlorophyll absorption in tomatoes. The chlorophyll content of ripening tomatoes decreased or disappeared completely. Due to the second overtone of the stretching of the O-H bond, there was a noticeable absorption peak at 969.72 nm [35].

### 3.3. Selection of Characteristic Wavelengths

**Figure 4:**
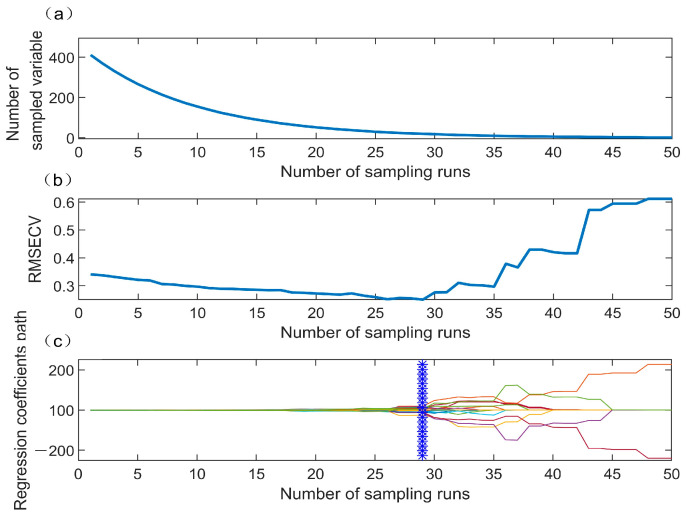
Selection of characteristic wavelengths by CARS. (**a**) The variation trend for wavelength number with the increase in sampling runs; (**b**) the variation trend of RMSECV; (**c**) the variation trend of the wavelength regression coefficient during variable selection. blue * indicates the optimal sampling number.

Selecting characteristic wavelengths can increase the operating speed and eliminate redundant information [36]. CARS was used to select the characteristic wavelengths. The number of MCS was set to 50 and five-fold cross-validation was utilized. The final selected wavelengths were determined by analyzing the values of the PLSR model. Figure 4 depicts the characteristic wavelength selection process of CARS. Figure 4a depicts a trend chart for wavelength number, reflecting the two different stages of effective wavelength selection: fast selection and refined selection. At the beginning, the number of selected wavelengths decreases sharply when the variables are forced out by EDF, and then the number of wavelengths becomes relatively stable. Figure 4b displays the RMSECV trend. During the first 29 sampling runs, the RMSECV of the PLSR model decreases slightly with the increase in the sampling times due to the gradual elimination of unimportant wavelengths. After the 29th sampling run, the RMSECV starts to become larger. Thus, the 29th sampling run represents the junction of underfitting and overfitting, and the subset of optimal variables. Figure 4c is a trend diagram of the wavelength regression coefficient, and the blue line indicates that the selected wavelength has the minimum RMSECV for the PLSR model when the optimal sampling number is 29. Finally, 20 wavelengths with distinct characteristics were chosen (518.57, 530.85, 555.54, 574.17, 595.39, 659.64, 662.18, 665.99, 671.07, 672.34, 686.33, 722.08, 736.17, 751.57, 782.45, 821.15, 898.76, 915.59, 935.00, and 984.13 nm).

### 3.4. Classification Model

**Figure 5:**
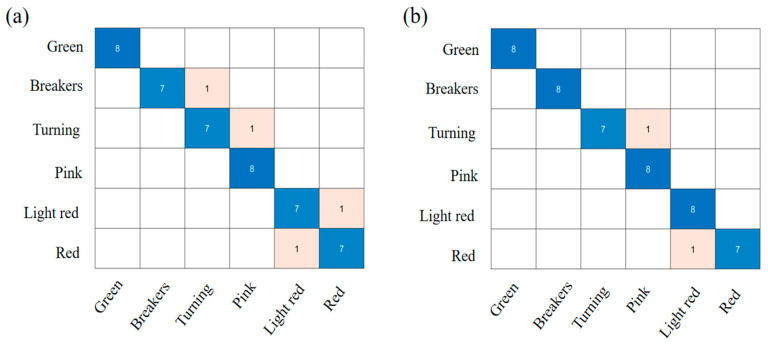
The confusion matrices of the prediction set with two different input data sets of SVC: (**a**) FULL-SVC and (**b**) CARS-SVC.

Randomly dividing 144 tomato samples into calibration sets and prediction sets with a ratio of 2:1, a total of 144 tomato samples were categorized. The tomato identification SVC models were established using the full (FULL) and characteristic wavelengths. Figure 5 depicts the confusion matrices of the prediction set with two different input data sets of SVC, while Table 2 displays the results of optimal parameters and the performance evaluation for SVC models based on FULL and characteristic spectral data.

As shown in Figure 5a, the prediction accuracy rate for the FULL-SVC model was 91.67%, and the precision, recall and F1-score were 91.90%, 91.67% and 91.79%. It was easy to distinguish between green and pink stages, whereas other stages were misclassified as adjacent grades. This may have been because the color distribution on the surface of the fruit was less variable on tomatoes of adjacent ripeness grades. Figure 5b showed the confusion matrices of the prediction set for the CARS-SVC model. The CARS-SVC model achieved a better performance of 95.83% accuracy rates, 96.30% precision, 95.83% recall, and a 96.07% F1-score. The classification accuracy of the pink and light red stages was higher than that of the breaker and turning stages, which is consistent with previous studies [37]. It was evident that the performance using the characteristic wavelength was higher than that using full wavelengths and that the number of feature variables was significantly lower for characteristic wavelengths than for that for full wavelengths. For modeling, it was necessary to extract characteristic wavelengths from full spectra. 

Cho et al. [38] developed a classification model for tomato maturity involving six stages using snapshot-type hyperspectral imaging and SVC. At the laboratory level, the highest classification accuracy and F1-score were 79% and 88%, respectively, while at the field level, classification accuracy and F1-scores were 75% and 86%, respectively. The main reasons for the low classification accuracy were that the tomatoes were grown in different periods and were affected by various test environments, such as camera angle, external light intensity, and so on. Nevertheless, hyperspectral imaging has the potential to be used as a useful tool to classify tomato maturity.

### 3.5. Quantitative Model

**Figure 6:**
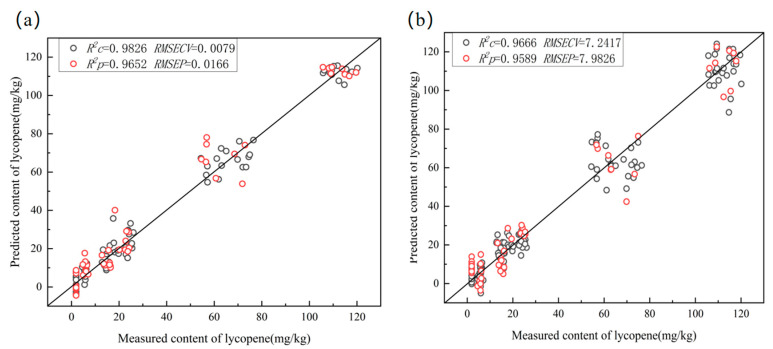
Predicted and measured lycopene content values for (**a**) SVR and (**b**) PLSR models based on characteristic wavelengths.

In total, 144 tomato samples were quantitatively analyzed. Briefly, 144 tomato samples were randomly divided into calibration sets and prediction sets with a ratio of 2:1. The SVR and PLS models were developed to quantitatively analyze tomato lycopene content based on the full and characteristic wavelengths. Choosing the appropriate parameters, c and g, is crucial for SVR to avoid over-fitting and improve modeling accuracy. The grid optimization algorithm search methods and five-fold cross-validation methods were used to optimize the parameters c and g. The five-fold cross-validation method was used to determine the optimal number of LVs. Table 3 provides the results of SVR and PLSR for predicting the tomato lycopene content in tomatoes. Models based on characteristic wavelengths performed significantly better than those based on complete wavelengths. In addition, it was observed that the SVR model exhibited excellent quantitative prediction capacity, regardless of whether it was based on the full wavelengths or the characteristic wavelengths, indicating that SVR possessed advantages in nonlinear small sample prediction. When the optimization parameters were c =64 and g = 0.0442, the CARS-SVR model yielded R^2^C = 0.9826, RMSECV = 0.0079 mg/kg, R^2^P = 0.9652, and RMSEP = 0.0166 mg/kg. Figure 6 illustrates the performance of the SVR and PLSR models by depicting the measured and predicted lycopene contents. A strong correlation can be observed between the predicted and measured values.

The results show that modeling with fewer wavelengths obtained based on feature selection does not only improve the predictive performance of the model, but also simplifies the model and improves its robustness. The established models, particularly the CARS-SVR model, can effectively detect the lycopene content of tomatoes. Rahman et al. [1] used hyperspectral imaging for the prediction of moisture content, pH and soluble solid content in intact tomatoes. The results showed that the best effect was obtained using PLSR with the correlation coefficients of 0.81, 0.69, and 0.74. These studies demonstrate that hyperspectral imaging has the potential to predict the chemical compositions in tomatoes nondestructively, which provides important guidance for determining the picking period, quality grading, transportation and storage of tomato fruits. 

### 3.6. Visualization of the Lycopene Content

**Figure 7:**
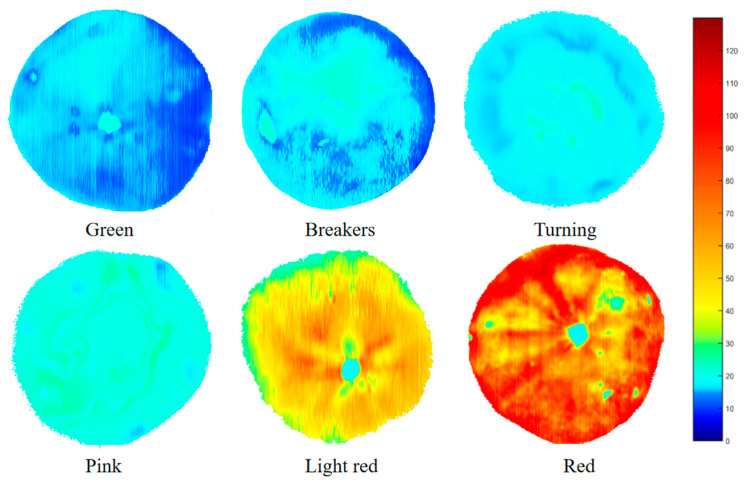
Visualization of lycopene content in tomatoes at green, breaker, turning, pink, light red and red stages.

To visualize the lycopene content of the tomatoes throughout their entire maturity stages, the CARS-SVR prediction model was applied and the calibration results of the multivariate analysis were transferred to each pixel of the hyperspectral image. Figure 7 depicts the visualization of the lycopene content. According to the color bar on the figure’s right side, blue indicated low content, red indicated high content, and the transition from blue to red indicated that the lycopene content of tomatoes gradually increased with maturity. The change in pseudocolors in Figure 2 corresponded to an increase in the lycopene content. When the lycopene content reached about 110 mg/kg, the whole tomato was close to red. As can be seen in Figure 7, the color of each part of the tomato was not regular, indicating that the distribution of the lycopene content in tomatoes was uneven. 

## 4. Conclusions

In this paper, hyperspectral imaging was applied to classify tomato maturity into six stages and to predict the lycopene content of tomatoes. SVC classification models were established using the full and characteristic wavelengths. The results showed that SVC classification models were established using the full and characteristic wavelengths. The results showed that the prediction set’s accuracy rate for CARS-SVC models was higher than that for FULL-SVC models, at 95.83%.

CARS established the SVR and PLSR models of the lycopene content based on the full and characteristic wavelengths. With R^2^P = 0.9652 and RMSEP = 0.0166 mg/kg, the models created by CARS-SVR yielded the best results regarding their effects and stability.

Following this, pseudocolors were generated to visualize the lycopene content of tomatoes. The result indicated that hyperspectral technology could detect tomato maturity during storage and quantitatively predict lycopene content. As a form of nondestructive testing technology, hyperspectral imaging technology can promote the development of intelligent tomato quality detection. In the study, only one main chemical component was predicted using hyperspectral imaging, and further research is needed to obtain more comprehensive information to comprehensively evaluate tomato quality.

## Figures and Tables

**Table 1:** The appearance description of tomatoes at different maturity stages.

Stage	Appearance Description
Green	The tomato’s exterior is entirely green
Breakers	The tomato’s exterior is entirely green. First appearance of pink or red exterior, covering not more than 10%
Turning	Above 10% but not more than 30% pink or red
Pink	Above 30% but not more than 60% pink or red
Light red	Above 60% but not more than 90% red
Red	Above 90% red

**Table 2:** The results of performance evaluation for SVC models based on FULL and characteristic spectral data.

Model	Variable Number	c	g	Accuracy of Calibration Set	Accuracy of Prediction Set	Precision	Recall	F1-Score
FULL-SVC	411	32	0.0156	93.75%	91.67%	91.90%	91.67%	91.79%
CARS-SVC	20	128	0.0313	97.92%	95.83%	96.30%	95.83%	96.07%

**Table 3:** The results of SVR and PLSR models for predicting the tomato lycopene content in tomatoes.

Model	Variable Number	c	g	LVs	Calibration		Prediction	
					R^2^_C_	RMSECV (mg/kg)	R^2^_P_	RMSEP (mg/kg)
FULL-SVR	411	2.8284	0.0221		0.9690	0.0155	0.9341	0.0305
FULL-PLSR	411			7	0.9330	10.2520	0.9236	10.9920
CARS-SVR	30	64	0.0442		0.9826	0.0079	0.9652	0.0166
CARS-PLSR	30			7	0.9666	7.2417	0.9589	7.9826

## Data Availability

Data are contained within the article.
